# Prescription Department

**Published:** 1894-09

**Authors:** 


NASAL CATARRH.

By Dr. D. M. Wick, New Hartford, Ia.:

℞ Eucalyptol (Sander & Sons) 30 minims.
Menthol crys., 1 gr.
Ol gaultheriæ, 3 minims.
Liq. vaseline, ℥ j. M.

Sig. Spray into nose as hot as can be borne.

TO HARDEN THE SKIN OF BEDRIDDEN PATIENTS IN ORDER TO PREVENT BED-SORES.

The parts first to be washed with an antiseptic solution twice daily, dry and apply the following powder:

℞ Pulv. camphor, ʒ v.

Amyl. pulv., ℥ iiiss.

Cretæ gallic, ℥ ij.

Alum ustum. ʒ j.

Acid boracic, ℥ j.

Pulv. oxid. zinc, ℥ j.

Acid carbolic, ʒ ss.

Oil of gaultheria, ʒ ss. M.

GONORRHEA.

Dr. Edward Martin, of Philadelphia, prescribes:

℞ Balsam capaiba,
Oil sandal-wood, āā m v.
Oil cinnamon, m j.

In capsule. Of these he gives 6 to 12 daily, administering them one hour after meals.

FOR EPILEPSY.

℞ Potassii bromidi, ʒ iv.

Tincturæ belladonnæ, f ʒ iij.

Infusi gentianæ compositus ad., f ℥ viij.

M. S. A tablespoonful thrice daily.

℞ Camphoræ monobromat, gr. xlviij.

Ext. gentianæ, q. s.

Ft. Massæ et div., in. pil. no. x.

S. One at bedtime.—BLACK in *Brit. Med. Jour.*

### ANTISEPTIC POWDER.

Dr. Albert Pick, of Boston, writes to the *New York Medical Journal* that he is in the habit of using, with very satisfactory results, a preparation thus composed:

℞ Hydrargyri chlorid. corros., gr., $\frac{1}{5}$–$\frac{1}{3}$.

Acidi borici, ℥ j.

Acidi tannici, gr. x.

Sacch. lact., q. s. ad ℥ ij.

M. Sig. Antiseptic powder.

A fifth of a grain of corrosive sublimate in this mixture gives a powder of the strength of 1 to 5000, and a third of a grain 1 to 3000.

### FOR AMENORRHEA.

℞ Hydrag. chlorid. corrosi, gr. $\frac{3}{4}$.
Sodii arseniat., gr. j.
Ferri sul. exsic., gr. xxx.
Potassii carb., gr. xv.
Ext. nucis vomicæ, gr. v.
M. Ft. pil. No. xxx.
S. One before each meal.—*Medical News*, from *Practitioner*.

### PERTUSSIS (WHOOPING COUGH).

℞ Ammonii, bromidi, ʒ ij.

Ex. chestnut leaves, fl. ℥ j.

Tr. belladonnæ, ʒ ss.

Syr. tolutani, ℥ ij.

Syr. simplicis, q. s. ad. ℥ iv. M.

Sig. One teaspoonful every two or three hours.

### TREATMENT OF GASTRO-INTERNAL CATARRH IN CHILDREN.

Demme, in the *Centralblatt fur die Gesammte Therapie*, recommends the following:

℞ Sodium paracresylate, gr. jss–iij.

Laudanum, ♏ jv.

Cognac, gtt. xv.

Simple syrup, ʒ jss.

Distilled water to make, ʒ j.

Sig. Teaspoonful every two hours.

